# Limbic grey matter changes in early Parkinson's disease

**DOI:** 10.1002/hbm.23610

**Published:** 2017-05-02

**Authors:** Xingfeng Li, Yue Xing, Stefan T Schwarz, Dorothee P. Auer

**Affiliations:** ^1^ Radiological Sciences, Division of Clinical Neuroscience, University of Nottingham, Queen's Medical Centre Nottingham NG7 2UH United Kingdom; ^2^ Sir Peter Mansfield Imaging Centre School of Medicine, University of Nottingham Nottingham NG7 2UH United Kingdom

**Keywords:** Parkinson's disease, amygdala, voxel‐based morphometry, structural connectivity, brain age‐related change

## Abstract

The purpose of this study was to investigate local and network‐related changes of limbic grey matter in early Parkinson's disease (PD) and their inter‐relation with non‐motor symptom severity. We applied voxel‐based morphometric methods in 538 T1 MRI images retrieved from the Parkinson's Progression Markers Initiative website. Grey matter densities and cross‐sectional estimates of age‐related grey matter change were compared between subjects with early PD (*n* = 366) and age‐matched healthy controls (*n* = 172) within a regression model, and associations of grey matter density with symptoms were investigated. Structural brain networks were obtained using covariance analysis seeded in regions showing grey matter abnormalities in PD subject group. Patients displayed focally reduced grey matter density in the right amygdala, which was present from the earliest stages of the disease without further advance in mild‐moderate disease stages. Right amygdala grey matter density showed negative correlation with autonomic dysfunction and positive with cognitive performance in patients, but no significant interrelations were found with anxiety scores. Patients with PD also demonstrated right amygdala structural disconnection with less structural connectivity of the right amygdala with the cerebellum and thalamus but increased covariance with bilateral temporal cortices compared with controls. Age‐related grey matter change was also increased in PD preferentially in the limbic system. In conclusion, detailed brain morphometry in a large group of early PD highlights predominant limbic grey matter deficits with stronger age associations compared with controls and associated altered structural connectivity pattern. This provides *in vivo* evidence for early limbic grey matter pathology and structural network changes that may reflect extranigral disease spread in PD. *Hum Brain Mapp 38:3566–3578, 2017*. © **2017 The Authors Human Brain Mapping Published by Wiley Periodicals, Inc.**

## INTRODUCTION

Parkinson's disease (PD) is the second‐most common neurodegenerative disorder, affecting up to 2% of individuals aged 65 years and older [Hanganu et al., 2014]. The hallmark motor symptoms (bradykinesia, rigor and tremor) are thought to share the same fundamental aetiology related to dopaminergic cell death in the substantia nigra pars compacta and subsequent reduction of striatal dopaminergic innervation [Brown, 2003]. The pathophysiology of non‐motor dysfunction is less well understood, and may reflect additional serotonergic, cholinergic and noradrenergic dysfunction. There is increasing recognition of the clinical relevance of these symptoms such as depression, apathy, fatigue, disturbed sleep, autonomic dysfunction and cognitive deficits.

Braak et al. [1994, 2003] showed relatively early involvement of the amygdala in post‐mortem studies, from stage 3 onwards in their postulated progression model of six consecutive disease stages affecting multiple neuronal systems. Disease progression with a prolonged premotor phase is clinically well established with many studies showing that a range of non‐motor signs precede the more typical motor manifestation of the disease [Iranzo et al., 2014; Kalaitzakis et al., 2013; Koller, 1992; Simuni and Sethi, 2008]. Remarkably, early symptoms are typically non‐motor, implying that Parkinsonism may not be the first manifestation of PD [Diederich et al., 2016; Klockgether, 2004]. Furthermore, retrospective studies indicate that affective symptoms may manifest as one of the first symptoms many years before the typical motor signs [Lemke, 2008]. Presence of non‐motor signs may also define a more severe disease subtype, for example, depression in patients with PD was found to be associated with a more rapid deterioration in cognitive and motor functions [Burn, 2002]. Studying brain regions associated with non‐motor symptoms in early stages of PD may help us to gain more insight into their pathophysiology and to unravel pre‐motor disease manifestation. Candidate regions that may be relevant for these non‐motor symptoms include limbic structures such as the hippocampus and the amygdala [Calabresi et al., 2013; Ricciardi et al., 2015; van Mierlo et al., 2015; Vriend et al., 2016]. Post‐mortem studies have shown a 20% volume loss of grey matter in the amygdala in PD [De la Monte et al., 1989; Harding et al., 2002]. Using regional MR volumetric methods, lower amygdala volume [Junqué et al., 2005] as well as hippocampal atrophy (as inferred from cross‐sectional data) [Camicioli et al., 2003] have been reported in some studies of patients with Parkinson's.

Whole‐brain voxel‐based morphometry (VBM) studies have failed to consistently confirm limbic grey matter changes. Interestingly, the two most recent VBM studies [Menke et al., 2014; Planetta et al., 2015] did not show any significant grey matter difference between patients with PD and healthy controls. This differs from earlier studies reporting significant grey matter volume reductions outside the limbic system, in the right superior temporal gyrus (BA 41, [Pereira et al., 2012]) or in the frontal lobe [Burton et al., 2004]. An earlier co‐ordinate‐based meta‐analysis suggested grey matter reductions in the right inferior frontal‐gyrus, extending to the superior temporal gyrus and the insula in patients with PD [Pan et al., 2012], but the method used lacked rigorous multiple test correction [Tench et al., 2013]. Several other studies found limbic grey matter abnormalities in specific subgroups of patients with Parkinson's and dementia, depression or anxiety. Notably, amygdala grey matter density loss was found in a number of studies using region of interest (ROI) analysis or whole‐brain VBM in specific subgroups [Bouchard et al., 2008; Diederich et al., 2016; Feldmann et al., 2008; Harding et al., 2002; Junqué et al., 2005; Ouchi et al., 1999; Surdhar et al., 2012; Tessitore et al., 2002; van Mierlo et al., 2015; Vriend et al., 2016]. Similarly, several studies reported hippocampal volume loss mainly in patients with cognitive impairment and PD dementia [Burton et al., 2004; Junque et al., 2005], while findings in PD without cognitive impairment reported inconsistent changes [Mak et al., 2015]. Taken together, morphometric studies did not reveal a consistent pattern of grey matter abnormalities in limbic structures or elsewhere in PD. The discrepancies between studies may be due to the limited power in many studies, but may also reflect true heterogeneity dependent on disease stage, medication state or subgroup effects. To overcome such limitations, we investigated a relative large number of homogenous early stage PD patients. This included a large proportion of non‐medicated patients to allow unbiased group comparison in hypothesis‐free VBM analysis.

An important advancement in the field of neuroimaging research is the study of network abnormalities in addition to local changes when using structural or functional brain imaging techniques. Functional connectivity studies are a rich source of information and become increasingly popular in the clinical neurosciences and PD [Buckner et al., 2013] despite several unsolved methodological issues [Bastos and Schoffelen, 2016]. Structural connectivity can be studied using diffusion tensor imaging (DTI) [Le Bihan et al., 2001], structural covariance analysis [Alexander‐Bloch et al., 2013; Chou et al., 2015; Mechelli et al., 2005] and independent component analysis. Interestingly, a recent structural connectivity study based on voxel deformation reported a basal ganglia structural network deficiency in PD in which the degree of GM loss was linked to motor severity [Chou et al., 2015]. More recently, large structural covariance network [Zeighami et al., 2015] and functional connectivity analyses [O'Callaghan et al., 2016] were successfully applied to characterise striatal and cerebellar network change in PD. No studies of PD related limbic structural connectivity alterations are available, despite the early pathophysiological involvement of the limbic system and the putative link with non‐motor symptoms. Moreover, although atrophy in amygdala and hippocampal regions have been investigated [Bouchard et al., 2008; Pereira et al., 2012], it is not clear whether this is a regionally specific finding or may reflect generally accelerated brain ageing, which would result in widespread increased cortical atrophy rates.

To address these knowledge gaps, detailed voxel and network based grey matter analyses were performed in a large MRI dataset from the PPMI database. First, we compared brain structural differences between healthy controls and PD patients using VBM. Second, correlations between amygdala grey matter intensity and clinical scores of non‐motor disease severity were investigated; we hypothesised that amygdala grey matter density loss would underpin non‐motor symptoms in PD. Third, we explored PD induced alterations of limbic structural connectivity using structural covariance analysis of the amygdala. Last, local age‐related grey matter density change was estimated in subjects with Parkinson's and compared with those estimated in healthy controls hypothesising that this may prove useful as a potential biomarker of PD progression.

## MATERIALS AND METHODS

### MRI and Clinical Data

Five hundred and seventy MRI T1 structural cross‐sectional images were used in this study initially. All MRI image data were obtained from the PPMI website (http://www.ppmi-info.org/) on 13/10/2015. The dataset included 178 healthy controls (113 male, aged 60.57 ± 11.38, mean ± standard deviation) and 392 people with Parkinson's (253 male, aged 62.09 ± 9.80). Six subjects from the healthy controls and 26 from the PD group were removed from the study due to grey matter segmentation problems. In the final analysis, 172 healthy controls (110 male) and 366 PD subjects (235 male) were included. Ages between different groups were not significantly different (*P* < 0.05). All clinical information including PD medication status (115/366 PD subjects), clinical severity and psychometry were also obtained from the PPMI database and included in the analysis (Table 1). More detailed information about the demographics and clinical data can be found on the PPMI website.

**Table 1:** Demographics and clinical details table (172 healthy controls and 366 PD subjects derived from PPMI repository)

	Healthy controls (mean ± SD)	PD (mean ± SD)
Age	60.62 ± 11.36	62.18 ± 9.80
Total MDS‐UPDRS I	4.68 ± 3.57	4.88 ± 3.30
Total MDS‐UPDRS II	7.93 ± 4.90	7.94 ± 4.76
Total MDS‐UPDRS III	**1.21 ± 2.18**	**21.60 ± 9.71**
Total GDS	4.11 ± 1.40	4.27 ± 1.55
Total ESS	6.61 ± 3.46	7.17 ± 4.13
HVLT	**49.72 ± 6.41**	**46.75 ± 7.72**
SCOP‐AUT (SCAU1∼SCAU21)	**4.83 ± 3.37**	**9.28 ± 6.73**
STAI state	**27.78 ± 8.89**	**33.60 ± 10.64**
STAI trait	**32.73 ± 6.40**	**35.24 ± 7.25**

Clinical information of healthy controls subject number 3063 could not be found and was thus discarded. Bold text in the table denotes a significant difference between healthy controls and people with PD (*P* < 0.01).

Abbreviations: ESS: epworth sleepiness scale; GDS: geriatric depression scale; HVLT: Hopkins verbal learning test; MDS‐UPDRS: Movement Disorders Society Unified Parkinson's Disease Rating Scale; SCOPA‐AUT: scale for outcomes in Parkinson's disease for autonomic symptoms; SD: standard deviation; STAI: state‐trait anxiety inventory.

To determine the effects of PD progression on grey matter morphometric changes, we aimed to classify the PD cohort according to severity, based on previously validated MDS‐UPDRS III severity thresholds [Martínez‐Martín *et al*., 2015]. We found there were only 26 (of 366) subjects who met classification criteria for moderate PD in the study group. Because of this, we instead used a median‐split to subgroup the PD patients into mild (MDS‐UPDRS part III < 21, *n* = 183) and mild to moderate PD (MDS‐UPDRS part III ≥ 21, *n* = 182). One PD subject was excluded in the clinical analysis due to missing MDS‐UPDRS III scores.

Several software packages and computer languages were employed in the analysis. The FMRIB software library (FSL) VBM package (http://fsl.fmrib.ox.ac.uk/fsl/fslwiki/FSLVBM) was adopted for image registration, image segmentation, grey matter modulation and image smoothing. Image Registration Toolkit (IRTK) (https://www.doc.ic.ac.uk/~dr/software/index.html) was also employed for image registration [Rueckert et al., 1999] if FSL failed for the image registration. R language (https://www.r-project.org/) was used for VBM analysis and statistical comparison between different groups. In addition, Python language (https://www.python.org/) was implemented to extract patient information from the clinical table, including search age, calculation of total MDS‐UPDRS and other clinical and psychometric scores. The results for each image and clinical data processing steps were checked visually.

### VBM Analysis

The FSL‐VBM processing was conducted as follows. First, structural T1 images were registered to the Montréal Neurological Institute (MNI) template using the FSL Linear Image Registration Tool (FLIRT) [Jenkinson and Smith, 2001] function. If the images failed to be registered, then the IRTK package with a manual registration was carried out to obtain the initial value for a rigid registration. A large head mask as part of the MNI template was employed to exclude shoulder and neck in the brain image. This was done by multiplying the registered images with the head mask using FSL‐maths functions. After that the brain extract tool (BET) method [Smith 2002] was employed to cut the skull from whole image for each of the 570 T1 MRI structural images. Next, non‐uniformity correction was carried out, and the FSL Automated Segmentation Tool (FAST v.4) [Zhang et al., 2001] was adopted to segment tissues according to their type. The segmented grey matter partial volume images were then aligned to the MNI standard space (MNI152) by applying the affine registration tool FLIRT (FMRIB's linear image registration tool) and nonlinear registration FNIRT (FMRIB's nonlinear image registration tool) methods, which use a B‐spline representation of the registration warp field. A study‐specific template was created by averaging the registered images (before smoothing), and used for non‐linear re‐registration of the native grey matter images. The registered grey matter partial volume images were modulated (to correct for local expansion or contraction) by dividing them by the Jacobian of the warp field. The segmented and modulated images were then smoothed with an isotropic Gaussian kernel with a standard deviation ( σ = 3mm). Based on the smoothed images, a GLM with permutation test was employed for the group comparison [Winkler et al., 2014]. All VBM comparisons between different groups were conducted within the framework of GLM. In the design matrix of GLM, subject age and sex were also included as covariates in the analysis. Cluster‐based threshold correction for the grey matter comparison was set to be *P* < 0.05. The significance threshold with the family‐wise error (FWE) was employed for the final significant statistical comparison. *T* statistical threshold (e.g., *t* > 4) was predefined for the FWE correction. To help localise grey matter differences, the 116 regions specified in the automated anatomical labelling (AAL) template [Tzourio‐Mazoyer et al., 2002] were used to label regions in the resulting statistical maps. Visual inspection was carried out in each step.

### Covariance Analysis

R language was adopted for the structural grey matter covariance analysis [Mechelli et al., 2005] to calculate the correlation coefficients between regional individual grey matter density (averaged within a predefined seed region) and the local grey matter density of the whole brain. In this study, we were interested in the structural connectivity of limbic areas affected by the disease and we thus selected limbic regions showing significant grey matter changes in PD vs healthy controls as seeds. Structural covariance analysis results in one correlation map per seed for the whole group. To enable between group comparison and testing for differences between healthy controls and patients with PD, we employed a linear regression model with group variables and interaction term as follows:

(1) Y=a+b⋅X+c⋅PD+d⋅PD⋅X+e

where *Y* is the averaged grey matter intensity in the seed region; *a*,*b*,*c*,*d* are the regression coefficients; *e* is the model error; PD is the disease state group variable with 0 for healthy controls and 1 for PD subjects; and *X* is the local grey matter intensity for structural covariance analysis. For each group, we normalised dependent variable *Y* and independent variable *X* separately. Then we solved the equation by using the least squares method. The coefficient *d* before the disease state and *X* is the interaction term; if *d* = 0, then there is no correlation difference between healthy controls and PD patients; if *d* is significantly different from 0, then there is a structural covariance difference between healthy controls and patients with PD. We applied *T* statistics to quantify the significance between different groups [Hocking, 2003]. To illustrate qualitative pattern differences within each group, we also applied a simplified within group regression model (e.g., *c* = 0 and *b* = 0).

### Age‐Related Grey Matter Intensity Change

To assess differences in age‐related grey matter intensity change between PD and control groups from a cross‐sectional sample, we undertook voxel‐wise estimation of age versus grey matter (GM) density separately in PD and control groups. We applied the same linear regression model as shown in Eq. (1) that allows us to compare local age‐related GM loss between healthy controls and PD patients. To achieve this, the dependent variable *Y* in this model denotes the local grey matter intensity and the independent variable *X* represents subject age with identical group. Again, we normalised grey matter intensity and age for each group separately. To test the deterioration speed difference between healthy controls and PD patients, *T* statistics were applied to assess whether coefficient *d* is significantly different from 0 or not. If *d* is significantly different from 0, it implies that there is a significant brain deterioration speed difference between healthy controls and PD patients.

For ROI analysis, we also calculated change speed for each group separately. This is achieved by simplifying Eq. (1), that is, set c=0 and d=0. Then in this reduced model, *b* denotes the brain age‐related grey matter density change and *X* represents the subject age and *Y* is the local grey matter intensity. Because *X* represents the subject age, regression slope *b* can be considered as an estimate of the predicted rate of grey matter degeneration [Ziegler et al., 2012] while rates of atrophy can only be measured using longitudinal data.

### Grey Matter and Clinical Data Correlation Analysis

In addition, correlation analysis was carried out to study the clinical relevance of observed limbic grey matter changes. To this end, we investigated the inter‐relation between clinical non‐motor scores and grey matter intensity in affected limbic areas. Similar correlations were investigated for grey matter intensity across abnormal limbic structural networks. Correlation between grey matter and clinical information was computed using *cor.R* function in R language. For each subject, because the clinical information was collected several times for many of the subjects, we used the clinical information which was the nearest to the MRI scan time. In some cases where there was a missing value in the clinical table, we replaced the value with most recent clinical information from the table. It should be mentioned that the VBM, covariance and structural connectivity analysis in this study was based on the grey matter intensity from the modulated and smoothed grey matter images in VBM analysis.

## RESULTS

### Grey Matter Density Loss in PD Primarily Affects the Amygdala

**Figure 1:**
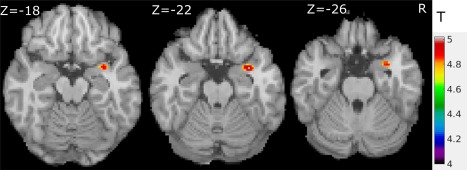
Grey matter intensity loss in PD (*n* = 366) versus healthy controls (*n* = 172) using FSL‐VBM with cluster‐based FWE corrected (*t* > 4, *P* < 0.05), seeing in the right amygdala region. [Color figure can be viewed at http://wileyonlinelibrary.com]

Using FSL‐VBM, we found few deficits in grey matter density in PD compared to healthy controls (PD < healthy controls) most notably in the right amygdala region (Fig. 1 and Table 2). Also, a small part of right superior temporal pole (Table 2) adjacent to the amygdala showed significantly reduced grey matter density in PD versus healthy controls. Because we used FWE with cluster‐based VBM for threshold correction for the group comparison, these results can be considered too conservative. This has been criticised and the risk of FWE to produce false‐negative results is well known [Bennett et al., 2009]. To validate the results, we hence applied R language to compare healthy controls and PD group using a less restrictive two sample *t*‐test method with an uncorrected threshold. In addition to the amygdala and superior temporal pole abnormalities, this showed significant (*T* > 2.8, *P* < 0.005, two‐tailed test) grey matter intensity loss in the following regions (Supporting Information, Fig. S1): parahippocampal (±27.4, 0.2, −30), hippocampal (−26.3, −3.2, −27.2), fusiform (35.3, −17.3, −34), pallidum (−23.2, −7.4, −6) and precentral cortex (−44.2, −11.2, 38). There were no regions with increased grey matter density in PD compared with healthy controls using both FWE threshold correction method and uncorrected methods.

**Table 2:** Grey matter density deficits in PD patients

Contrast groups	Brain regions	MNI coordinates (*x*,*y*,*z*)	*T* value
All PD patients (*n* = 366) < healthy controls (*n* = 172)			
	Right amygdala	(31, 1.6, −18)	4.58
	Right superior temporal pole	(31.3, 5.2, −21.1)	4.15
Mild (MDS‐UPDRS3 < 21) (*n* = 183) < healthy controls (*n* = 172)			
	Right amygdala	(28.5, 1.3, −27.8)	4.40
	Left amygdala	(−29.2, 1.3, −27.6)	4.60
	Left middle temporal pole	(−29, 17.7, −37.1)	4.41
	Right fusiform gyrus	(34.9, −8, −38.9)	4.03
	Right superior temporal pole	(32.8, 4.3, −22.1)	4.69
	Parahippocampal region	(−28.2, 1.6, −29.9)	4.16

### Effect of Disease Severity on Grey Matter Density

**Figure 2:**
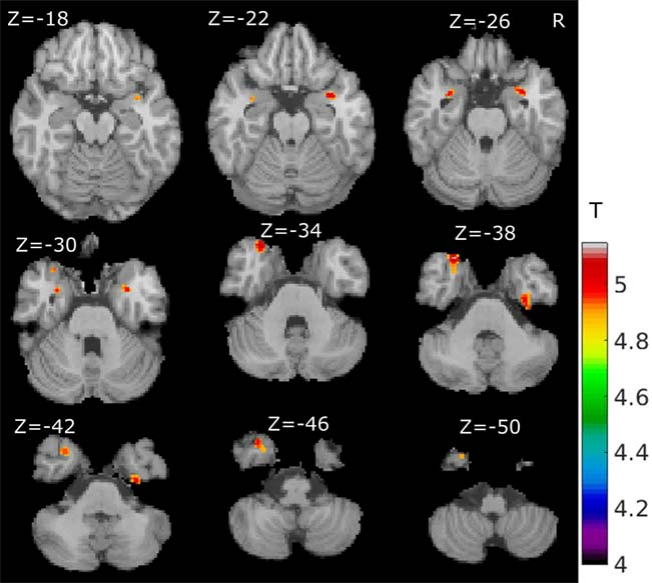
Grey matter intensity loss in mild PD (total MDS‐UPDRS III score < 21, 183 subjects) versus healthy controls (*n* = 172) with age and sex as covariates (FWE correction, *t* > 4, *P* < 0.05). [Color figure can be viewed at http://wileyonlinelibrary.com]

We compared 172 healthy controls with 183 mild MDS‐UPDRS‐III < 21 and 172 healthy controls with 182 mild‐moderate subjects (MDS‐UPDRS‐III ≥ 21). Again, though age was not significantly different between the mild and mild‐moderate subgroups, we included age and sex as covariates in the design matrix of the GLM for the two subgroups compared with healthy controls. Using the same statistical threshold and correction method in VBM analysis as in healthy controls versus PD comparison (Fig. 1; 172 healthy controls versus 183 mild PD comparison), we found a significant difference in grey matter density in the bilateral amygdala, left middle temporal pole, right fusiform gyrus, right superior temporal pole and parahippocampal region as shown in Fig. 2 and Table 2.

For the comparison of 172 healthy controls versus the remainder 182 mild‐moderate PD group, we found similar regional differences as in Fig. 1 (results not shown here). However, the significant region was much smaller; for the healthy controls versus all PD patients comparison, 77 voxels survived after FWE correction, whereas only 27 survived for the healthy controls versus mild‐moderate PD comparison. This surprising result cannot be explained by medication effects, as a similarly low number of subjects were on dopaminergic medication (51 [27.9%] in mild PD and 64 [35.2%] in mild‐moderate PD). We did not find statistical differences between the subgroups of 183 mild MDS‐UPDRS‐III < 21 PD and 182 mild‐moderate PD patients using VBM.

### Non‐Motor Symptoms and Amygdala GM

Cognitive impairment was significantly correlated with lower right amygdala GM (HVTL: *R* = 0.155, *P* = 0.033 and UPDRS 1.1 *R* = −0.111, *P* = 0.03). Also the severity autonomic dysfunction (SCOPA‐AUT) was significantly larger with lower right amygdala GM density. By contrast, we did not find associations with anxiety scores.

Owing to the possible confounding effects of age and sex on amygdala GM density, we repeated the association using multivariate regression controlling for age and sex. This confirmed an independent significant association of SCOPA‐AUT (*T* < −2.79, *P* < 0.006) and HVLT (*T* > 3.99, *P* < 0.0001).

### Structural Connectivity Deficits in the Limbic System in PD

**Figure 3:**
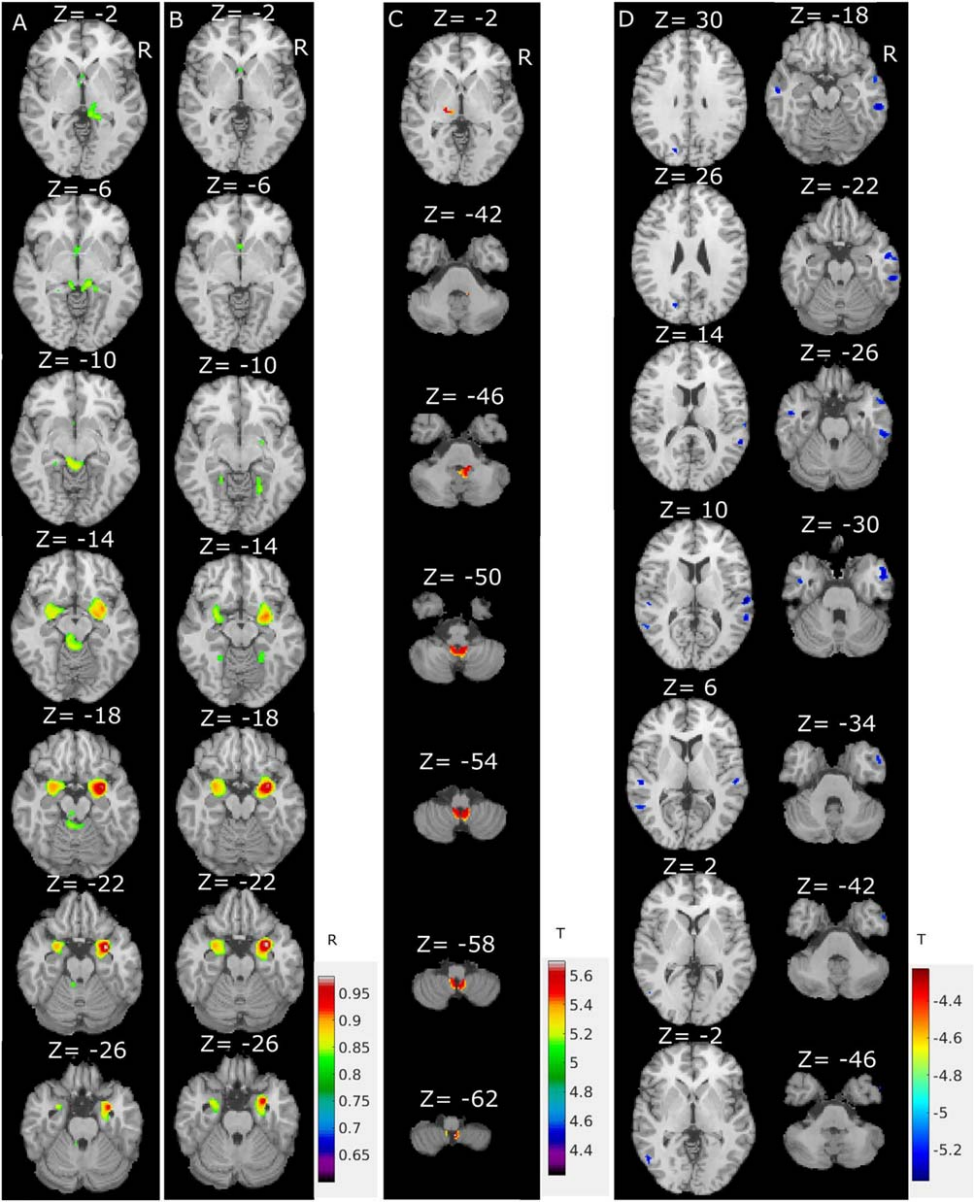
Structural connectivity analysis to right amygdala grey matter seed shows reduced and altered covariance in PD. (**A**) Healthy controls group covariance map (*n* = 172). (B) PD group covariance map (*n* = 172). (C) Structural connectivity difference map: healthy controls > PD, FWE corrected (*P* < 0.05). (D) Structural connectivity difference map: healthy controls < PD, FWE corrected (*P* < 0.05). [Color figure can be viewed at http://wileyonlinelibrary.com]

The averaged grey matter intensity within the right amygdala seed region (extracted from VBM result, see Fig. 1) and the whole brain grey matter intensity was then calculated using the simple within group regression model (Fig. 3A,B). To allow an unbiased group comparison, we used the same number of healthy controls and PD subjects, and thus randomly selected the 172 (the total number of healthy controls) subjects from PD group, when calculating the correlation maps for healthy controls (Fig. 3A) and PD (Fig. 3B) groups. The mean age of the healthy controls was 60.62 ± 11.36 years, which did not differ from the 172 PD group (61.59 ± 10.11). Groups were also well matched for sex (110 males in controls and 112 males in patients).

In Figure 3A,B, the colour regions denote the significant correlation false discovery rate correction (*P* < 6 × 10^−8^) between the affected amygdala region and the whole brain. A small *P* value was adopted, thresholding the correlation map. Similar results were found when using the rest of the PD subjects (366 − 172 = 194) as a confirmatory correlation test. Furthermore, we repeated the random selection and correlation calculation processing 100 times, and then averaged the correlation from these 100 random samples. The results (not shown here) were very similar to the correlation analysis results using all PD subjects as shown in Figure 3B. In the healthy controls, we found structural connectivity of the right amygdala with the left amygdala, hippocampus (±29.3, −8.6, −14), and parahippocampus (±25.4, −28.2, −14), brainstem, thalamus (±15.8, −14.7, 10) and superior cerebellar vermis. In contrast, the structural network in PD was limited to the amygdala, parahippocampus and fornix, suggesting long‐range structural connectivity deficits in PD.

In addition, Eq. (1) was employed to test for significant group differences. The structural amygdala network showed significant differences between the PD and healthy control groups with reduced covariance in the cerebellum and thalamus regions (Fig. 3C and Table 3), but stronger covariance in bilateral temporal cortices and to a lesser extent the left occipital cortex (Fig. 3D and Table 3) in patients.

**Table 3:** Structural right amygdala covariance changes in PD

Brain regions	MNI coordinates (*x*,*y*,*z*)	*T* value
Healthy control > PD		
Left cerebellum	(−3.7, −49.8, −52.1)	5.33
Right cerebellum	(6.7, −50, −52.1)	4.79
Right thalamus	(12.7, −20.7, −1.4)	4.76
Healthy control < PD		
Left superior temporal gyrus	(−58.8, −24.2, 7.8)	−5.51
Right superior temporal gyrus	(54, −27.1, 7.8)	−4.81
Middle temporal gyrus	(55.9, −55.7, 7.8)	−4.59
Middle temporal gyrus	(−61.6, −42.4, 7.8)	−4.54
Left inferior temporal gyrus	(−55.9, 29, −25.1)	−4.63
Right inferior temporal gyrus	(51.1, −5.1, −27.6)	−4.30
Right superior occipital	(19.6, −78.5, 25.4)	−4.39

### Increased Age‐Related Grey Matter Intensity Reduction in PD Preferentially Affects the Limbic and Paralimbic System

**Figure 4:**
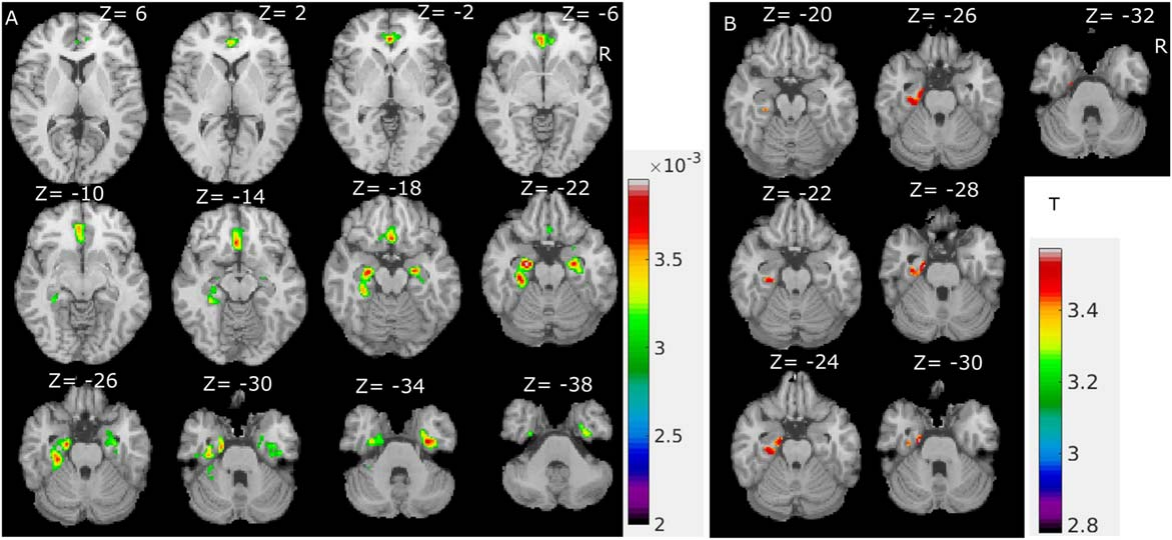
Maps of increased age‐related changes in PD. (A) Between group difference (PD age‐related change − healthy control age‐related change) shown at a cut‐off of 0.002/year. (B) Statistical comparison, significant age‐related change difference map (PD age‐related change > healthy control age‐related change); FDR threshold correction (*P* < 0.05). [Color figure can be viewed at http://wileyonlinelibrary.com]

Maps of age‐related grey matter intensity change were calculated for both PD and healthy control groups using Eq. (1) with c=0 and d=0 to obtain voxel‐wise age‐related change (age predictor regression slope) b for each group separately. We then subtracted the control groups' age‐related GM change maps from those in PD group to yield a difference map that represents the local increase of age‐related change in PD. The difference map was thresholded at two thousandths/year (absolute age‐related change difference) as shown in Figure 4A. Figure 4A shows the difference results with cluster size threshold of 20 voxels using 26 neighbourhoods. The largest regional grey matter change differences are summarised in Table 4. No areas with a negative grey matter change difference were found at the threshold of two thousandths/year.

**Table 4:** Brain regions showing a bigger age‐related change in PD compared with healthy controls

Brain regions	MNI coordinates (*x*,*y*,*z*)	Change speed difference ( 000/year)
Right hippocampus	(23.6, −10.8, −18)	3.2
Left hippocampus	(−23.6, −10.8, −18)	2.96
Left parahippocampus	(−26.7, −9.5, −34)	2.43
Rectus gyrus	(−1.5, 23.4, −18)	3.19
Fusiform gyrus	(−28, −34.3, −18)	2.93
Frontal middle orbital gyrus	(−4.1, 40.7, −10)	3.13
Anterior cingulum	(0.7, 41.2, 2)	2.86
Temporal middle lobe	(50.5, −56.8, 14)	3.47

Because the biggest alterations were seen in core limbic hubs, hippocampus and parahippocampus regions (Fig. 4A), we applied post hoc ROI analysis to compare the difference between PD and healthy controls in these regions.

For each region, the right hemispheres and left hemispheres are compared separately as shown in the supplement (Supporting Information, Fig. S2 and Table S1). Regression results showed that the regression slopes of all these regions are significantly steeper in PD, confirming that the right parahippocampus (Supporting Information, E of Fig. S2) has the largest increase in age‐related GM change in PD followed by the right hippocampus and right amygdala (Supporting Information, Table S1).

Similar to covariance group comparison, we applied Eq. (1) to test for significant deterioration speed differences between patients and controls. As shown in B of Fig. S2, we found significantly increased age‐related grey matter intensity change in patients with Parkinson's in the left parahippocampal region (−29, −22.5, −24.6, *T* = 3.12) and a small cluster in the left fusiform gyrus (−32.8, −23.7, −25.1, *T* = 3.13) using small volume and false discovery rate (FDR) threshold correction (*P* < 0.05).

## DISCUSSION

In this study including 538 subjects, we compared the structural grey matter density, connectivity, and age‐related grey matter intensity change estimates between healthy controls and early PD. The main findings are predominant right‐hemispheric limbic grey matter alterations in early PD, as demonstrated by right amygdala grey matter deficits and reduced cerebellar, but increased temporal connectivity and increased age‐related grey matter intensity changes in the right limbic and paralimbic system.

### Preferential Amygdala Grey Matter Intensity Decrease in Early PD

VBM analysis of this large dataset of brain MRI revealed that the amygdala was the most prominently affected grey matter region in PD subjects. This result is in good agreement with previous post‐mortem [Harding et al., 2002] and some MRI studies [Junqué et al., 2005] which showed amygdala volume reduction. Although numerous previous papers report amygdala deficits in PD subjects [Bouchard et al., 2008; Burton et al., 2004; Vriend et al., 2016], amygdala atrophy is mainly seen in subgroups of PD patients with dementia [Junqué et al., 2005] or mood disorders [Diederich et al., 2016] and mostly reported in patients with more advanced disease. Also amygdala changes in those studies are not the predominant finding. Interestingly, we found right amygdala grey matter density loss as the most consistent abnormality in a large group of early PD patients, 93% classified as mild [Martínez‐Martín et al., 2015] (MDS‐UPDRS‐III < 33) and 69% not on PD medication. We confirmed strong amygdala grey matter intensity loss also in the more mildly affected subgroup of patients with MDS‐UPDRS‐III < 21 (Fig. 2). This finding provides further evidence that there is relatively early involvement of the amygdala in PD subjects [Diederich et al., 2016].

### Amygdala Grey Matter Change and Non‐Motor Symptoms in Early PD

In this sample, we found partial support for a role of amygdala degeneration and non‐motor symptoms in early PD, specifically cognition and autonomous dysfunction but not anxiety. We showed significant (*P* < 0.5) negative correlation between right amygdala grey matter intensity and SCOP‐AUT score. A positive significant correlation between amygdala grey matter intensity and HVLT was also discovered and confirmed by a negative correlation with the cognitive impairment UPDRS I subscore. Moreover, we show that these associations are independent of age and sex effects. Despite the low correlation strengths, our findings extend previous reports on a cognitive role of the amygdala in patients with Parkinson's even in early stages when only mild cognitive abnormalities are noted. It is however noteworthy that we did not observe an interrelation between right amygdala GM density and anxiety scores in contrast to several previous studies [Remy et al., 2005; Wen et al., 2016]. This may be explained by the mildly elevated anxiety scores that may not have been severe enough to reveal associations with amygdala deterioration. Our findings are however in line with a recent VBM study in PD [Vriend et al., 2016] that reported no association with amygdala volumes and total Beck anxiety score. Interestingly, they found lower amygdala volumes with higher ‘psychological’ anxiety subscores. Moreover, the interrelationship between anxiety and limbic morphometry is complex, with some studies reporting increased amygdala volumes in generalised anxiety disorders [Schienle et al., 2011], and both children and adults with high trait anxiety [Qin et al., 2014]. More detailed studies including analysis of amygdala subnuclei in well phenotyped longitudinal cohorts are required to clarify the neuroanatomical basis of anxiety dimensions in PD.

To the best of our knowledge, we report the first investigation of a link between autonomic dysfunction and amygdala grey matter reduction in Parkinson's. Patients with higher scores of autonomic dysfunction showed lower amygdala grey matter density. This observed association is well supported by the anatomical connections of the amygdala with major projections to regulatory centres of the autonomic nervous system. The amygdala forms part of the central autonomic network [Beissner et al., 2013] overlapping the salience network (bilateral insula and midcingulate). Our findings are broadly in line with a suggested role of the central autonomous network in cardiac dysautonomic function in PD. A recent small study [Chen et al., 2016] in 23 patients with PD showed associations between hippocampal and insular volumes and baroreflex sensitivity, and also reported reduced right amygdala volumes, but did not find associations between amygdala volume and baroreflex sensitivity. More detailed analyses of dysautonomic subscores are warranted to further assess the nature and possible symptom and regional specificity of the central autonomic network dysfunction in PD.

### Structural Amygdala Covariance is Disrupted in PD

We studied the structural connectivity between amygdala with other brain regions to explore whether early amygdala involvement could trigger disease progression across a structural network centred on the amygdala. Using seed‐based covariance analysis [Mechelli et al., 2005], we found stronger structural covariance between the amygdala and cerebellum and posterior thalamic nuclei in the healthy control group. Connections and projections from the amygdala to the thalamus have been demonstrated in animal studies [Aggleton and Mishkin, 1984; Su and Bentivoglio, 1990] and are supported by functional connectivity studies using resting‐state fMRI [Roy et al., 2009]. Pathways from the thalamus to the amygdala are important in emotional learning [Pessoa and Adolphs, 2010]. To the best of our knowledge, no detailed grey matter structural covariance analysis has been reported for the amygdala. The observed structural covariance at similar thresholds (*Z* > 2.3, data not shown) overlaps with the functional amygdala network, as derived from resting‐state fMRI data including the insula, thalamus, cingulate and medial prefrontal cortex (in Fig. 2 of Roy et al. [2009]).

Intriguingly, we observed an altered long‐range structural covariance network of the right amygdala in Parkinson's patients. There were fewer connections to the cerebellum and left thalamus, but increased connections were noted to bilateral temporal gyri and left occipital cortex in patients with PD. The observed disconnection of the right amygdala is of particular interest in view of the disconnection from cerebellar regions, as this connection has been shown to reflect an important brain ‘alarm system’ for subliminal signals of fear [Liddell et al., 2005]. In the healthy control group, the amygdala handles this ‘alarm system’ efficiently but subconsciously and rapidly; however, if the network is disrupted, then the reaction time in response to an emotion stimulus will be increased [Tessitore et al., 2002]. We noted additional increased connections of the right amygdala with bilateral temporal association cortex pointing to network alteration that either reflects compensatory processes or within network synchronisation of disease‐related grey matter modulation. On the basis of primary observation of amygdala grey matter deficit, we consider the second interpretation as more likely despite absence of temporal cortical deterioration evidence from VBM in this cohort. Covariance analysis can be considered more sensitive, and in fact some previous VBM studies reported temporal lobe abnormality in Parkinson's [Camicioli et al., 2003]. Regardless of the nature of the shown network alterations, the changes imply that the emotion processing network is affected in early PD in line with the amygdala as a densely connected ‘hub’, coordinating and integrating tasks and providing a general handling of emotion. This may provide an explanation for the emotional face recognition difficulties in PD that could however not be probed in this patient sample [Diederich et al., 2016].

### Increased Limbic Age‐Related Grey Matter Change in PD

We investigated age‐related grey matter change in PD subjects as a further approach to explore the pattern of disease progression. Brain atrophy in PD subjects has been recognised for a long time [Bouchard et al., 2008; Camicioli et al., 2003; Junqué et al., 2005]; however, most of these studies focus on the regional grey matter changes in the hippocampus and amygdala of PD. Because previous studies did not employ voxel‐based methods to study age‐related GM change, they did not allow characterisation of the temporal‐spatial pattern of brain ageing in PD. To address this, we applied a linear regression method and used cross‐sectional age information to map local age‐related grey matter change [Ziegler, et al., 2012]. This method based on cross‐sectional data does not allow to infer on atrophy but provides a means to estimate and compare age‐related grey matter change between groups. Nevertheless, such group‐based estimates of age‐related rates of GM loss are indices of predicted atrophy rates. However, our results are in general good agreement with previous work, demonstrating that the hippocampus, parahippocampal regions and parts of the amygdala showed a stronger age‐related grey matter deficit in PD versus controls (Table 4), with strongest increase in age‐related GM change in the right hippocampal region (for healthy controls, the predicted age‐related GM loss rate is −0.001234; for PD patient group, the rate is −0.004434). Taken together, we describe an extended limbic and frontotemporal pattern of augmented GM loss in early PD cases suggesting that extrastriatal deterioration is a region‐specific and earlier phenomenon than previously considered.

### Strengths and Limitations

One of the advantages in this study is that we combined different software packages and languages for optimal data analysis, and employed Python to process text files and string variables from clinical information tables. We applied R language for the VBM, structural covariance and statistical analysis to compare different subject groups. We combined IRTK and FSL software packages for image registration. The other advantage of this study was that we employed the relatively large PPMI data with comprehensive clinical information. Most previous studies used a smaller number of subjects (<200 subjects). It should be pointed out that PPMI data is from multi‐centres (33 centres from different countries) with different scanners introducing technical heterogeneity, yet, overall, multi‐centre structural MRI [Stonnington et al., 2008] studies are thought to have greater statistical power than single‐centre studies for VBM analysis [Schnack et al., 2010]. Our study has however several limitations. Foremost, it was based on cross‐sectional structural MRI image analysis, and thus estimates of age‐related grey matter change was based on different subjects, precluding direct assessment of atrophy rates. To overcome this limitation, future studies are needed using serial MRI to evaluate the effects of individual brain ageing more accurately. Last, the clinical and psychometric scores indexing non‐motor symptoms were limited not allowing to study more detailed structure–symptom relationships. The PPMI is an early cohort of patients and we did not observe mood or sleep abnormalities at group level preventing meaningful correlation analyses. There are also some missing values in the cognitive measures. Importantly, the relation between grey matter intensity and these measures may not be linear, and while grey matter changes will be stable, variability of the clinical measures even within 1 day may have further masked undetected associations. Nevertheless, the large sample size allowed us to detect significant albeit weak associations between amygdala GM reduction and some non‐motor symptoms at the early stages of PD.

Further research is warranted to more fully investigate putative region and network‐specific limbic atrophy patterns underpinning specific non‐motor symptoms, such as specific autonomic dysfunctions, psychological versus somatic affective symptoms, olfactory and face recognition impairment.

## CONCLUSION

This study provides new evidence for amygdala involvement, associated structural disconnection, and increased medial temporal age‐related grey matter change in early PD based on advanced morphometric analyses in a large sample of patients. These findings support the early affection of the limbic network in Parkinson's and a role in autonomic dysfunction and early cognitive impairment, and offer novel tools to track extranigral disease progression.

## FUNDING

This work was supported by Parkinson's UK [grant number J‐1204].

## CONFLICT of INTEREST

The authors declare no competing financial interests.
